# Diet-based strategies, informed by genetics, to improve healthspan

**DOI:** 10.1093/geroni/igaa057.3200

**Published:** 2020-12-16

**Authors:** Sean Curran

**Affiliations:** Leonard Davis School of Gerontology, University of Southern California, Los Angeles, California, United States

## Abstract

Diet is one of the most variable aspects of life history, as most individuals have a large diversity of choices, varying in the type and amount that they ingest. In the short-term, diet can affect metabolism and energy levels. However, in the long run, the net deficiency or excess of calories from diet can influence the progression and severity of age-related diseases. An old and yet still debated question is: how do specific dietary choices impact health- and lifespan? It is clear that genetics can play a critical role - perhaps just as important as diet choices. For example, poor diet in combination with genetic susceptibility can lead to metabolic disorders, such as obesity and type 2 diabetes. We have identified the existence of diet-gene pairs, where the consequence of mutating a specific gene is only realized on specific diets. Although only a handful of these diet-gene pairs have been characterized, there are potentially thousands of such interactions, which may explain the variability in the rates of aging in humans and the incidence and severity of age-related diseases.

